# Older adults make sense of their suicidal behavior: a Swedish interview study

**DOI:** 10.3389/fpsyt.2024.1450683

**Published:** 2024-09-03

**Authors:** Sara Hed, Anne Ingeborg Berg, Stefan Wiktorsson, Jennifer Strand, Silvia Sara Canetto, Margda Waern

**Affiliations:** ^1^ Department of Psychiatry, Institute of Neuroscience and Physiology, University of Gothenburg, Gothenburg, Sweden; ^2^ Department of Neuropsychiatry, Sahlgrenska University Hospital, Region Västra Götaland, Gothenburg, Sweden; ^3^ Department of Psychology, University of Gothenburg, Gothenburg, Sweden; ^4^ Department of Psychotic Disorders, Sahlgrenska University Hospital, Region Västra Götaland, Gothenburg, Sweden; ^5^ Department of Psychology, Colorado State University, Fort Collins, CO, United States

**Keywords:** older adults, nonfatal suicidal behavior, despair, interview study, interpretative phenomenological analysis, geriatric psychiatry

## Abstract

**Introduction:**

The aim of this study was to explore how individuals aged 70 or older living in Sweden understood a recent suicidal act, and what changed in them and around them in the aftermath.

**Method:**

Four women and five men (age range 71-91 years) receiving care at a geriatric psychiatric outpatient clinic in a large Swedish city took part in two interviews about their most recent suicidal act. Most of the women and none of the men had engaged in prior suicidal acts. Interpretative phenomenological analysis was employed.

**Results:**

The suicidal act was explained as a response to losses (in physical and cognitive functions, social roles and relationships) that rendered previous coping strategies unviable. The participants reported being dependent on a healthcare system that they experienced as indifferent and even dismissive of their suffering. The suicidal act was described as an unplanned act of despair. Positive changes followed for participants who reported having had suicidal ideation prior to the suicidal act and had insights into its triggers. Some gained access to needed medical care; others developed greater awareness of their psychological needs and became more effective at coping. Individuals who said that they had not had suicidal thoughts prior to the suicidal act and could not explain it reported no positive change in the aftermath. The respondents’ narratives indicated gendered themes.

**Discussion:**

Participants’ age-related losses were in many cases exacerbated by negative interactions with health care providers, indicating that continued attention needs to be given to implicit ageism in medical professionals. The suicidal acts were described as impulsive, which was unexpected because a dominant belief is that older adult suicidal behavior is planned. One reason for the discrepancy may be that this study focused on nonfatal acts, and planned acts may be more likely to be fatal. Another reason could be shame due to suicide stigma. Alternatively, these acts were truly unplanned. The older adult suicide planning question should be addressed in larger studies across geographical and cultural settings. Future studies should also include questions about gender norms of suicidality and separately examine women’s and men’s data.

## Introduction

Around the world nonfatal suicidal behavior is the most prevalent form of suicidality (1). Globally, the combined prevalence of nonfatal and fatal suicidal behavior among older adults is high (1). A paradox of suicidality, including among older adults, is that women are less likely to die of suicide than men, but women are more likely to be diagnosed with depression and have higher or similar rates of nonfatal suicidal behavior, relative to men (2). While the number of suicidal acts per suicide death is lower among older adults than among other age groups (3), a previous suicidal act is a strong predictor of death by suicide in older adults (4). Yet older adults are underrepresented in research on nonfatal suicidal behavior (5).

Quantitative studies, primarily conducted in high-income countries, have identified factors associated with nonfatal suicidal behavior. Among these are mental illness, including both major and minor depression and problematic alcohol use, previous suicidal behavior, physical illness and disability, loss of autonomy, low level of education, relationship problems and social disconnectedness, financial difficulties, and personality (e.g., 6–12). In recent years there has been increasing research on the link between mild cognitive impairment (MCI) and both suicidal ideation (13) and behavior (14).

There seems to be a discrepancy between the risk factors reported in quantitative studies and the older adults’ own narratives of their suicidal behavior. The authors of a qualitative study conducted in England (15); concluded that the risk factors identified in the suicide literature were often absent or viewed by older adults who survived a suicidal act as irrelevant to their suicidal act. Including the study by Crocker and colleagues, we could identify only four qualitative studies involving interviews with older adults following an act of suicidal behavior (15–18). The findings of these studies were reviewed in two studies (19, 11). Themes summarized in these reviews included loss of control, loneliness and isolation, impaired decision-making, accumulated suffering and pain, threats to identity, lacking reasons to live, ageing-related challenges in daily life, and feeling like a burden. Only two of the reviewed studies involved interviews with older adults in psychiatric care (16, 18). Both of these studies focused primarily on the events and states of mind preceding the suicidal act. Questions not addressed are how older adults integrate their suicidal behavior in their personal story and how they anticipate managing a future suicidal crisis, if they were to face one. Increased understanding of these phenomena could inform the development of clinical interventions that target suicidal older adults.

Building on and extending prior interview research with older adults who survived a suicidal act, this study explored how individuals aged 70 or older living in Sweden understood a recent suicidal act, and what changed in them and around them in the aftermath.

## Materials and methods

### Participants and procedure

Individuals living in a large Swedish city and receiving outpatient care at a geriatric psychiatric outpatient clinic following a suicidal act were invited to participate in the study. Inclusion criteria were age 70 years or older (corresponding to the minimum age for referral to the clinic); a suicidal act within the past 3-36 months; being capable of giving written informed consent and participation approval from their psychiatrist. Exclusion criteria were a clinical diagnosis of dementia or a Montreal Cognitive Assessment (MoCA) score of less than -2 standard deviations from Swedish normative score for age and education level (20); a personality disorder, active psychosis, delirium, or aphasia diagnosis; and insufficient knowledge of the Swedish language. Persons deemed by their psychiatrist as being at high risk of suicide were also excluded. Both women and men were included in the current study as rates of nonfatal suicidal behavior are rather similar in women (46/100 000) and men (52/100 000) aged 65+ living in Sweden (21). Since women live longer than men, the actual numbers of nonfatal acts are nearly identical in women and men (21).

Twenty-two individuals were identified by the clinic’s staff as meeting the study’s criteria. These individuals were asked if they would like to receive information about the study. Thirteen declined to receive information and to participate in the study. Reasons included a desire to move past the suicidal episode; life circumstances; lack of energy; or limited time primarily due to somatic illnesses and frequent medical visits. The individuals who accepted the invitation (n=9) received oral and written information about the study, including information about the interview (i.e., that the interview would focus on their most recent suicidal act and its aftermath), and were given the opportunity to ask questions about the study before giving a written consent. Each participant was interviewed twice. Prior to the interviews, a research psychologist (SW) carried out a psychiatric assessment, including a cognitive screening, to ensure that all participants met study criteria. All participants completed the study protocol which involved a psychiatric assessment and two interviews. Recruitment started August 2021 and ended December 2022.

The study sample included four women and five men (age range 71-91 years, median 76 years) (See **Table 1** for participants’ demographic profile, mental health and suicidality history). The time between the interview and the suicidal act ranged from 4 to 27 months. Eight of the participants were born in Sweden and one had immigrated from central Europe in early adulthood. MoCa scores ranged from 23-29 (mean 25.6); two of the participants met criteria for Mild Cognitive Impairment (MCI). The participants’ Montgomery-Åsberg Depression Rating Scale (MADRS: 22) scores ranged from 0-33, median 12. The female and male participants differed in terms of demographic characteristics, mental health and suicidality history. The women were either divorced or widowed, and living alone. All men but one were married. Half of the women were elementary school-educated; half were college-educated. One man had a college degree, the other men were high school educated. Most of the women and none of the men had engaged in prior suicidal acts.

**Table 1:** Demographic characteristics, mental health and suicidal behavior history of older adults who engaged in a nonfatal suicidal act.

Participant pseudonyms	Age	Sex	History of psychiatric care	Previous suicidal acts (SA)^a^	Age at first SA	Health care provider at the time of index SA	AD^b^at index SA	Preparatory acts at index SA (C-SSRS)^c^	Method^e^ at index SA, Level of lethality (C-SSRS)^f^	Clinical diagnosis after index SA	Months between index SA and interview	Depression^g^ at the time of the research interview
Anna	74	F	None	Yes	20	Primary care	Yes	No	Self-poisoning 4. Severe physical damage	Mixed anxiety and depressive disorder, Alcohol dependence	7	No symptoms
Jane	76	F	Recurrent contact since her 20s, no ongoing contact	Yes	61	Primary care	Yes	No	Self-poisoning 2. Moderate physical damage	Bipolar disorder, depressive phase Alcohol dependence	4	Mild depression
Emma	80	F	Recurrent contact since her 30s but no ongoing contact	Yes	35	Primary care	Yes	No	Jumping from height Aborted SA 0. No physical damage	Bipolar disorder, manic phase	27	Moderate depression
Cecilia	91	F	Brief contact in her 20s	No	91	Primary care	Yes	Yes^d^ (Note with a single word)	Self-poisoning 3. Moderately severe physical damage	Major depressive disorder, recurrent. Sleep disorder	6	No symptoms
Ben	71	M	Brief contact in his 40s	No	70	Primary care	Yes	No	Self-poisoning 1. Minor physical damage	Bipolar disorder, depressive phase	14	No symptoms
Tom	75	M	None	No	73	Primary care	Yes	No	Hanging 1. Minor physical damage	Major depressive disorder, recurrent	16	Moderate depression
Otto	75	M	Sporadic contact in later life but not ongoing	No	75	Primary care	No	Yes (Brief phone text to spouse)	Self-poisoning 3. Moderately severe physical damage	Major depressive disorder, single episode	5	Moderate depression
Sam	76	M	None	No	76	Primary care	Yes	No	Self-poisoning 3. Moderately severe physical damage	Major depressive disorder, single episode. Hypochondrical disorder	4	Moderate depression
Johan	81	M	None	No	80	Primary care	Yes	No	Self-poisoning 3. Moderately severe physical damage	Major depressive disorder, single episode	12	No symptoms

^a^SA, Suicidal act; ^b^AD, Antidepressant; ^c^C-SSRS, Columbia Suicide Severity Rating Scale, Item: Preparatory acts or behavior = Acts or preparation toward imminently making a suicide attempt. This can include anything beyond a verbalization or thought, such as assembling a specific method (e.g., buying pills, purchasing a gun) or preparing for one’s death by suicide (e.g., giving things away, writing a suicide note). ^d^ The medical record reports a note (a single word) not reported in the interview. ^e^ All self-poisoning involved own prescribed psychotropic medication. A few acts combined prescribed psychotropics with over-the-counter pain medication or drugs prescribed for their medical conditions. ^f^C-SSRS item: Actual lethality/medical damage = 0. No physical damage or very minor physical damage (e.g., surface scratches). 1. Minor physical damage (e.g., lethargic speech; first-degree burns; mild bleeding; sprains). 2. Moderate physical damage; medical attention needed (e.g., conscious but sleepy, somewhat responsive; second-degree burns; bleeding of major vessel). 3. Moderately severe physical damage; medical hospitalization and likely intensive care required (e.g., comatose with reflexes intact; third-degree burns less than 20% of body; extensive blood loss but can recover; major fractures). 4. Severe physical damage; medical hospitalization with intensive care required (e.g., comatose without reflexes; third-degree burns over 20% of body; extensive blood loss with unstable vital signs; major damage to a vital area). 5. Death. g. Level of depression based on MADRS score (Montgomery-Åsberg Depression Rating Scale).

As documented in **Table 1**, none of the participants was receiving psychiatric care at the time of the suicidal act although several had been in contact with psychiatric services in the past. Two men had received a short period of psychotherapy before their non-fatal suicidal act, but this contact was no longer ongoing at the time of the act. All participants had been prescribed antidepressants by their general practitioner prior to the suicidal act; one had discontinued taking the antidepressant due to side effects. Information about psychoactive prescription medication before and after the suicidal act and at the time of the research interview is shown in **Supplementary Table 1**. After the suicidal act and ensuing hospital medical care, seven individuals were referred to the geriatric psychiatric inpatient ward. The other two were referred to geriatric psychiatric outpatient services.

### Interviews

Each participant took part in two interviews with a geriatric psychologist (SH, this article’s first author) one to two weeks apart. This procedure was chosen because of the age of the participants and also because of the time required by the interview and the sensitive information to be covered. Our previous experiences interviewing older adults after a nonfatal suicidal act suggested that having some structure in the interview is important when discussing sensitive topics in this age group (18). A semi-structured interview guide was developed based on previous research on suicidal behavior in older adults. This approach provided a certain degree of structure while allowing each participant’s personal story to unfold and lead the course of the interview (23). The opening question “Can you tell me how you reached the point of attempting suicide?” was followed by questions such as “What triggered and intensified your plans and actions?”

Both interviews focused on the most recent suicidal act. The first interview included questions about interactions with relatives and care professionals before and after the suicidal act. In the second interview emphasis was placed on events related to the suicidal act and how the individual was coping. The two-interview method enabled the researchers to plan for the second interview based on the first interview.

Five participants chose to meet the interviewer at the geriatric psychiatry outpatient clinic, and four were interviewed in their own homes. The interviews (n=18) were conducted in Swedish and lasted between 45 and 90 minutes. The interviews were audio recorded and transcribed.

### Analysis

The Interpretative-Phenomenological-Analysis method (IPA: 24) was used to develop the interview and to analyze the interviews. IPA was chosen because it is designed to understand how people make sense of an experience. IPA’s epistemological positions are phenomenology and hermeneutics (24). The phenomenological approach is embedded in the research and interview questions, reflecting our intention to capture subjective sense-making processes of the suicidal act. The hermeneutic stance was mainly present during the analysis as the intention was to understand the participants’ making sense of their own experiences, thus creating a double hermeneutic. The ontological position adhered to was critical realism, which acknowledges that people’s understanding is always subjective and influenced by context.

In line with the IPA steps described by Smith and colleagues (2022), each interview was ideographically analyzed. Two of the authors (SH and AIB) read each interview transcript and made separate descriptive (phenomenological) coding. Thereafter, SH and AIB reread the interview transcripts and separately made interpretative (hermeneutic) notes. Emergent themes in each transcript were identified and connections between themes were documented. SH and AIB then compared their coding and discussed links between themes. Thereafter, each case was discussed with authors SH, AIB, SW, and MW, all of whom had read the transcripts and taken notes. This discussion focused on how participants narrated their suicidal act in their individual context. Similarities and differences among cases were explored. During the analytic process the authors iteratively returned to the transcripts to check on themes and to ensure that the themes were consistent with the participants’ narratives. Finally, a thematic structure consisting of four superordinate themes with subthemes was developed and agreed on by all authors. The analyses continued through the writing process. During the entire analysis, the intention was to integrate the participants’ and the researchers’ interpretations of the suicidal act and its aftermath.

### Ethics and reflexivity

The study was approved by the Gothenburg Regional Ethics Committee (2020-00321). During the project, the Swedish team worked closely with the clinical staff of the institutions where the sample was recruited. All participants had regular mental health care at the geriatric psychiatry clinic, whom they could turn to before, during, or after participation in the study. The participants were assured that they could withdraw from the study at any point and that the findings would be made available to them upon the study’s completion. SH, the interviewer, was employed at the geriatric psychiatric clinic where the data were collected and has experience working with depressed and suicidal older adults. SH’s clinical experience was helpful in handling the sensitive nature of the topics discussed in the interview, as some participants experienced mental fatigue and a temporary increase in anxiety during the interview. The interview’s pace was flexible. Participants were offered breaks and the option to continue the interview at another time. The study’s protocol stipulated that the participant’s psychiatrist would be notified immediately if suicidal feelings were to be triggered during the study. This was, however, not necessary because no participant reported suicidal ideation during or after the interviews. Several participants spontaneously said the interviews had been a positive experience. The participants’ well-being was always a priority during the interview and as a result, the length and content of the interviews varied. Pseudonyms were used to protect the identities of the respondents.

The research team was dominated by professionals with clinical interests and experiences. There was a concern that the interviewer’s clinical interests and experiences would influence the participants’ narratives. To counteract this, open-ended interview questions were used and follow-up questions were always based on the participants’ responses. The authors’ clinical pre-understanding was also in focus during the analyses. The transcripts were re-read several times to make sure that codes, notes, and themes were grounded in data, consistent with the qualitative research practice of investigator triangulation. The different professional and personal profile (e.g., discipline, seniority, cultural) of the co-authors broadened the analyses’ lenses within and between cases. In a final step, the research team’s cultural and gender expert (SSC) provided extensive cultural and gender feedback about the results and contributed to expanding the cultural and gender framework of the method and discussion.

## Results

**Figure 1:**
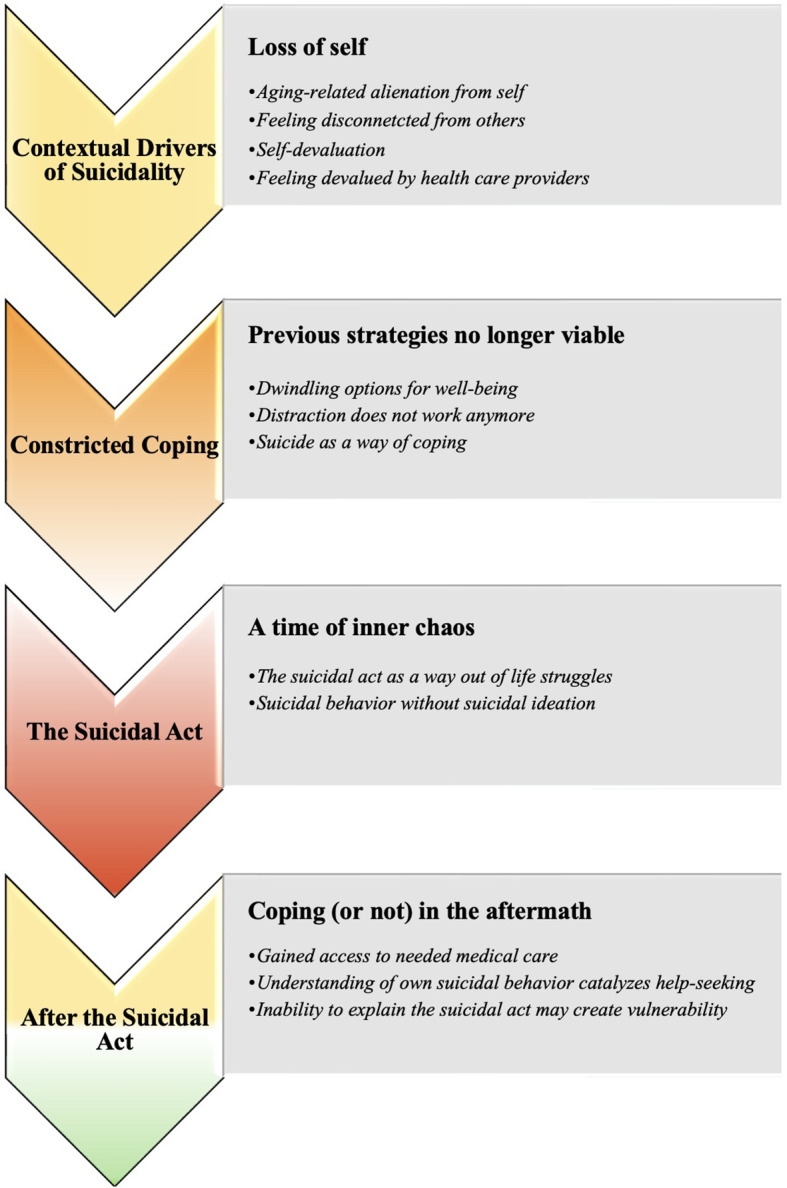
How older adults made sense of their own suicidal behavior: Superordinate and subordinate themes.

Themes generated by our analyses are organized in a temporal structure, from the events preceding the suicidal act to thoughts and situations experienced after the suicidal act (**Figure 1**). The superordinate themes are: 1) Contextual drivers of suicidality: Loss of self, 2) Constricted coping: Previous strategies no longer viable, 3) The suicidal act: A time of inner chaos, 4) After the suicidal act: Coping (or not) in the aftermath. Each superordinate theme has 2 to 4 subthemes (see **Figure 1**).

### Contextual drivers of suicidality: loss of self

Participants described age-related losses (e.g., losses in physical capabilities, social engagements and independence) that they were unprepared for and found distressing. Some said that they had tried to ignore aging-related limitations. A sense of isolation was described by many. Some participants reported that prior to their suicidal act they lacked knowledge about mental illness and about mental-health care options. In one way or another, all participants said that they had lost valued aspects of their identity.

#### Aging-related alienation from self

Alienation from oneself was described as a suicide driver. Several participants said that losses in relationships, health, and physical or cognitive decline triggered a loss of identity. The participants said that they did not recognize themselves and struggled to cope with the physical limitations they experienced and the challenges of accepting and adapting to changed conditions. An inability to contribute to others and society triggered a sense of loss in purpose and meaning.

Throughout her adult life, Cecilia had been in charge of herself. She had abundant energy and had dedicated herself to caring for others. In her early nineties Cecilia experienced episodes of confusion that impacted on her ability to carry out daily tasks. This led to feelings of inadequacy followed by rumination. Cecilia said that she felt that she was failing her loved ones. The loss of the ability to care for others represented for her an existential crisis.

…the thought, that I had made a huge mistake, it embedded itself deep within my mind, and I just couldn’t handle it. I don’t understand (…) it was as if I had lost connection with myself entirely. (Cecilia, 91 years)

Cecilia’s account suggests an experience of helplessness that brought on feelings of shame and a subjective loss of value as a person. She did not view her feelings of worthlessness as a mental health issue. The thought of seeking mental health care did not occur to her prior to the suicidal act.

Being assertive and getting things done had been a salient part of Otto’s identity. He had felt valued and appreciated for those qualities. Otto talked about how age-related losses had stifled his drive to have a public impact, and what that meant for him as a man.

A man perhaps needs to assert himself more, or has a need to create something, whether it is to build a house, plant a tree or write a book. Men have that drive to manifest their presence in the world. (…) I’ve felt some kind of pride, … sometimes when I’ve written something that I think is good. It is something that I’ve created that didn’t exist in the world before and that … that means something to me (…). I think that men have more of a need to find some kind of … justification for … their right to live. (Otto, 75 years)

The loss of opportunities to assert himself and to accomplish things he would be admired for was experienced as an existential threat by Otto.

#### Feeling disconnected from others

Many participants said that feeling disconnected from others had preceded the suicidal act. Feeling disconnected was described as a sense of standing alone in the face of challenging circumstances and as a sense of abandonment. Some talked about a feeling of not belonging. For some participants the loneliness followed the loss of a loved one. Others described profound loneliness despite having relationships.

Jane had always seen herself as a loner. Still, after the death of her husband, she felt a new sense of loneliness, and coping with life’s challenges became increasingly difficult.

The loss that fell over me when I didn’t have all this practical stuff to deal with, when it started to calm down (after the funeral). Then I actually started to realize … I don’t have my husband anymore. (…) I can’t live on my own. Alone.” (Jane, 76 years)

Johan recounted how loneliness had become overwhelming. Advancing in age, he experienced the loss of close relationships, including the passing of his partner. Intensified by the pandemic, this loneliness meant an abundance of time for rumination. His inner monologue became fraught with negative existential questions.

It is the loneliness that is the worst for me. I go into myself somehow. And then the (suicidal) thoughts come. What am I doing here? (Johan, 81 years)

#### Self-devaluation

Several participants said that they ruminated over failures. All who reported ruminating exhibited moderate depression symptoms at the time of the interviews.

Emma believed that her recurrent depressions made it difficult for others to be with her. She said that she had always been self-critical. Over time, she felt that she had become even more self-critical and that she came to believe that she did not deserve to live.

I think I have finished living. I’m tired of this. I don’t think highly of myself (…) and really, I’m quite a failure if you look at the whole life span. (Emma, 80 years)

Otto described how age-related losses led to changes in his thoughts. Things that had previously brought him pride became an occasion for negative rumination.

Some of those thoughts are very much self-accusations and a kind of anger and questioning of the choices I had actually made. (Otto, 75 years)

#### Feeling devalued by health care providers

All participants discussed how their age-related physical losses increased their dependence on the health care system, and all described having had negative interactions with care providers. They expressed that their psychological distress and life-weariness were met with a lack of response from both primary care doctors and specialists alike, which made it difficult to seek help. Some reported that their interactions with the healthcare system had heightened their hopelessness and left them to believe no help was available. For some participants these experiences triggered suicidal ideation.

Anna suffered from a severely disabling condition, the consequence of an obstetric complication that worsened with age. This condition made her feel embarrassed so she started to withdraw from social life. The operation that would have resolved her condition was not treated as urgent by her medical providers, and then postponed because of the pandemic. Anna lost confidence in the health care system. She felt dismissed and unworthy of living.

My doctor said it would take three years. (…) and I said, in three years I won’t be alive. That’s how it felt then. (…) I mean, you can wait three months. But three years. You know, I’m old, so three years is a very long time for me. (Anna, 74 years)

Johan, a man with frequent medical care contacts due to multimorbidity, said that the way health care providers related to him was crucial to his suicidal act. He said that he had never thought of taking his own life until he was discharged from the hospital with very short notice. He believed that the discharge decision was inappropriate and based on an incorrect assessment of his condition.

The thought of killing myself, it came just then, when the doctor let me down. Then there was nothing more, it was enough. So, then I did it. (Johan, 81 years)

### Constricted coping: previous strategies no longer viable

All participants described how their well-being had diminished. They also said that their coping options had become more limited and that coping strategies they had used when they were younger were no longer viable. For some, thoughts of death then started to provide comfort.

#### Dwindling options for well-being

Participants expressed that as older adults they had fewer paths to well-being as previous coping outlets were reduced. They felt lost when difficulties arose, as they discovered that their previous means of coping no longer worked.

Cecilia had always used baking to feel good about herself and to care for the ones she loved. At the age of 91, she continued to make cakes and cookies and she wanted to be there for everyone, as she had been in the past. Yet baking now made her feel exhausted.

Sam described a challenging upbringing but was determined to leave it behind and live a full life. He described himself as a lifelong hypochondriac. As he aged, his somatic concerns became more frequent and harder to treat, making it increasingly difficult to find relief. He felt guilty about contacting his health care providers so often. Shortly before he self-poisoned he read the doctor’s notes and felt rejected by the negative tone of the notes.

I constantly visit the primary care center (…), I’ve been there often, unfortunately. But then they wrote about this (his excessive consultations and hypochondria) in my medical record online. I didn’t know that they always write (their notes) on the internet. (…) I was so saddened by what I read. (Sam, 76 years)

Otto reported that age-related losses in physical capacities reduced his autonomy and sense of freedom, ultimately resulting in depression. He said that when he was younger, he tackled challenges by actively altering the course of his life when things were not as he desired. He explained that age-related losses in physical vitality deprived him of an important way to deal with anxiety and mental pain.

Throughout life, I have always felt a power to be able to take hold of things when things come to a standstill. A feeling that I could grab it. If I had been younger, I could have started over like that. I have no real opportunity to do that anymore. (Otto, 75 years)

Otto vividly remembered what it was like to feel physically and mentally strong, to make changes, and to accomplish things. He was painfully aware that his vigorous days were over.

#### Distraction does not work anymore

All participants said that in the past they had used distraction to deal with difficult circumstances but that distraction was no longer effective. They also said that the loss of distraction as a coping mechanism contributed to their suicidal act.

Otto could no longer engage in things that mattered to him. He described his unsuccessful attempts to distract himself from anxiety by reading or listening the radio.

So, the anxiety lies quite close under the surface. And one way to deal with that anxiety is to just flip through a newspaper, start a book, listen to the radio. Sometimes I find myself doing it all at the same time, I turn on the radio and I open a book, and … in that pattern I see a desperation. (Otto, 75 years)

Anna used alcohol as a distraction and a way to cope while waiting for needed surgery. Over the years, her alcohol consumption increased to the point of becoming an addiction.

The alcohol was something I resorted to in order to cope in some way. Alcohol numbs emotions somehow. (…) But it was too much. (Anna, 74 years)

Anna said that she experienced an overwhelming sense of being unwanted and unworthy. Eventually alcohol was no longer effective as a distraction. One day she called up a relative and admitted that she was an alcoholic. Within days of that phone call, she engaged in a suicidal act.

#### Suicide as a way of coping

Three different patterns were evident regarding thoughts of death and suicide. Some participants could not recall thoughts of death or suicide prior to the suicidal act. Others said that prior to the act they had thoughts of death but not of suicide. Yet others reported suicide thoughts prior to the suicidal act.

Sam, Ben, and Cecilia reported feeling hopeless, fatigued, resigned and desperate. They also said that that had not experienced life-weariness or death wishes before their suicidal act. The idea of suicide was so alien to them that they avoided speaking about their suicidal act with words like suicidal thoughts and suicidal act.

Tom and Anna remembered having had thoughts of death before their suicidal act. They recalled wishing to die quickly and painlessly. However, they did not recall having had thoughts of suicide prior to the act.

Johan had no previous history of suicidal thoughts but reported having had suicidal thoughts in short proximity of his suicidal act. Emma, Otto, and Jane had experienced suicidal thoughts earlier in life and talked openly about death and suicide. They said that thoughts of suicide and euthanasia offered them a mental escape when anxiety and depression became overwhelming. Suicide felt like an option, and a relief, when the other coping strategies did not work.

I remember carrying it with me like a kind of … comfort or I don’t know which word is quite right. Comfort or … consolation, the possibility that, even if it gets even more damn hard, if it gets even darker, then I get out. …so, it gave a certain relief … It sounds paradoxical, but it gave a kind of hopefulness … There was still a way out. (Otto, 75 years)

Emma described experiencing an almost constant wish to die. Because she had survived multiple suicidal acts, she came to doubt her ability to take her own life. Therefore, she found herself thinking about euthanasia.

I have thought that out in detail … We go down (to Switzerland), both the children and the grandchildren. (…). We check in to a hotel, and then we have a good time there for an afternoon and an evening. And then the next day… (Emma, 80 years)

Emma explained how the idea of euthanasia made her feel more at ease, implying that she could avoid guilt and stigma if a physician prescribed the fatal dose.

### The suicidal act: a time of inner chaos

All participants characterized the suicidal act as impulsive. None reported planning. As shown in **Table 1**, only one participant reported communicating their suicidal intention. This individual texted his spouse as he embarked on the act. Participants said that they experienced a sense of detachment during the suicidal act, an inability to think rationally, and a disregard for the outcomes and consequences of the act. From the participants’ accounts, two patterns became apparent. For some suicide came to represent a way out of life struggles. For others the suicidal act was unexplainable.

#### The suicidal act as a way out of life struggles

This theme captures the experiences of those who acknowledged having had either death and/or suicidal ideation before the suicidal act. In their chaotic state, suicide appeared to be the only solution to escape their life challenges.

After feeling wronged by the health care professionals, Johan said that suicidal ideation came abruptly. He recalled having one thought - that he wanted to escape it all.

In that situation, I’m so confused that I don’t know what I’m thinking about - I don’t have any anchor points then. (…). Then there was only one thought, ….I don’t want to be here. There was nothing more to do, this was the solution I could come up with. (Johan, 81 years)

Emma remembered feeling unbearable pain in her leg. She was unable to get a hold of the home care service. Feeling desperate, she climbed onto the windowsill of her sixth-floor apartment.

I didn’t want to jump to my death, I never even thought that far. But maybe there was something there, in the back of my mind. It wasn’t planned (…) But desperate. Because I mean, if you stand on the sixth floor and say you’re going to jump, there’s only death down there, I suppose, (…). I simply didn’t think about the consequences. The only thing I wanted was someone to come and help me. (Emma, 80 years)

Emma said that the suicidal ideation that she had experienced in connection with her most recent suicidal act was different from the suicidal ideation of her prior acts. Her past suicidal acts had involved a conscious decision to kill herself, whereas the most recent suicidal act occurred in a chaotic state of confusion and despair.

#### Suicidal behavior without suicidal ideation

Some participants said that their suicidal act was not preceded by ideation. They described feeling confused and without comprehension of their behavior. It “just happened.” They also said that they could not recall the details surrounding their suicidal act. These participants appeared to experience a lack of agency regarding their suicidal act. They said that they were “not themselves”, and that the act was incomprehensible to them. These individuals also said that they had felt overwhelmed by unbearable emotions, an emotional chaos. They described the suicidal state as a state of mental breakdown.

Tom’s recollection of the suicidal act was that he had acted on an impulse, and that the impulsivity frightened him. Tom realized that his act could have been lethal. What prevented his from killing himself is that the knot he had made in the rope did not hold.

I didn’t think about the consequences at all. It was absolutely insane. The idea was just to check the height and stuff like that (the rope). But why do you do that (try to hang yourself)? Because I really had no intention of doing it at the time. But it was so stupid … It’s like I said … it’s all a bit hazy. (Tom, 75 years)

Sam said that his suicide act was unplanned. He reported having no suicidal thoughts before the act. He described that he had felt sad, invalided and rejected. He maintained that he was not on his right mind when he took the tablets that could have killed him. Given his long-time health anxiety and fear of death, he could not explain why he had engaged in a suicidal act.

I don’t remember, I just took those damn tablets. I took a huge glass of booze too. I had a bottle ….at home, I took it (…). It was such a strange feeling; I don’t know why. It’s the opposite, I’m worried about my health. And I now think a lot, why did I do that thing, terrible. Why did I do it? (Sam, 76 years)

### After the suicidal act: coping (or not) in the aftermath

Some respondents talked about how the suicidal act changed their relationships with health care providers and family members. For some, there were also shifts in self-awareness, which influenced their ability to seek help. Those who engaged in a suicidal act without ideation struggled the most in the aftermath because they felt clueless and helpless about the act they had engaged in.

#### Gained access to needed medical care

Several participants gained access to the medical care they needed after receiving psychiatric care following their suicidal act. Some related that their suicidal ideation resolved once their medical care needs were taken seriously.

Anna believed that the hospitalization at the psychiatric ward following the suicidal act facilitated her access to the surgery she had sought for a long time.

I probably only got the operation because the doctor (psychiatrist) wrote something to someone … well, I don’t know what they wrote, but I think they influenced it. (…) I felt … they took me seriously. After all, no one had done that before. (Anna, 74 years)

After the surgical procedure Anna reduced her alcohol intake and experienced no further suicidal ideation.

Ben told a similar story. In the aftermath of his suicidal act, he met a psychiatrist who initiated psychotropic medication appropriate for his condition. This experience restored Ben’s trust in the healthcare system. Ben said he had learned that good professional support was available. He said that he would seek professional help if he encountered another suicidal crisis.

#### Understanding of own suicidal behavior catalyzes help-seeking

For those who explained their suicidal behavior as a response to a life problem and made changes to alleviate that problem, the suicidal act contributed to increased self-understanding. These individuals also developed a greater awareness of their own needs.

Johan recalled a suicidal ideation episode that occurred several months after his suicidal act. When the senior centre closed due to the pandemic, he experienced loneliness and isolation. Eventually, his thinking took a positive turn.

I sat there and the walls were staring at me. It was a pandemic, everything was closed. This is not good for me. I can’t sit here. Since I have thought about it before and made an attempt, the thought came quickly when I was in that situation. I felt no reason to continue living. Just because my life was sitting on the sofa … I wasn’t allowed to go out, not to take the bus, not do anything … So, it was like, the world had shrunk. (Johan, 81 years)

This time Johan did not try to handle the problem alone. He engaged in conversations with his children and with care professionals at the geriatric psychiatry clinic. Johan reported that the suicidal act had enabled him to better recognize and understand his own needs, which made it possible for him to adapt to new conditions and make important life changes. He said that he had learned that social connections played a vital role in his well-being and started acting in ways more congruent with his social needs.

Some participants talked about changing their perspective on suicide as it became apparent that their suicide would have negatively impacted their loved ones. For Otto, seeing his wife’s reaction after his suicidal act made him realize that his suicide would have hurt her.

This feeling about relief was so enticing it overshadowed it … I didn’t think through realistically what it would mean for other people. (…) Because I can’t do this, to my wife in particular. So, like that, the door has been closed. It’s like I’ve decided that I can’t do it, I can’t do it and … and that thought should have existed earlier… (Otto, 75 years)

#### Inability to explain the suicidal act may create vulnerability

Those who engaged in a suicidal act without awareness of prior suicidal ideation struggled to make sense of their suicidal behavior. Several said that they were not themselves or described themselves from the outside, distancing from their suicidal act by attributing it to their illness, or by minimizing the seriousness of the act and described it as impulsive.

Cecilia explained that she could not, and never would, understand why she had engaged in a suicidal act.

This happened … and I’ll never understand … and I can’t understand … I cannot imagine anyone being able to understand that something like that happens to people. (Cecilia, 91 years)

For Sam, the question of why he engaged in a suicidal act occupied his thoughts, causing anxiety.

I regret it something terribly, now. That’s the worst. Why did I do that? I go and ruminate. Since I am so afraid of death and of diseases, why did I do it? That’s the only thing I can think of. Why did I do that? (Sam, 76 years)

After the suicidal act, Sam lived in fear of having inflicted long-term damage upon himself.

## Discussion

In this study we aimed to explore how individuals aged 70 or older understand their suicidal act and the changes that followed. A first set of findings is that the participants described their suicidal behavior as a response to feelings of alienation from oneself and from others, following aging-related losses (i.e., in physical and cognitive functions, social roles and relationships) that rendered their previous means of coping no longer viable. These findings are consistent with a diversity of evidence that late-life suicidal behavior often occurs in the context of aging-related losses (e.g., 25, 26). The participants expressed despair over their dependence on a healthcare system that they perceived as indifferent and dismissive of their suffering. Their experience could be construed as a lack of visibility. The latter, defined as “feeling invisible or disconnected from others” (15, p. 638) also emerged as a theme in the qualitative study by Crocker and colleagues. As older people are more dependent on the health care system, the quality of the interactions with health professionals may be particularly influential for well-being.

Participants in our study described a lack of response from primary care and specialist physicians concerning information and coordination of care as well as unhelpful responses to their feelings of life-weariness and other psychological concerns. They were left with a belief that there was no help available, which heightened their sense of hopelessness. In a few cases the participant said that such hopelessness triggered the suicidal act.

A second set of findings is that the suicidal act was described as impulsive. The participants experienced the suicidal state as a time of mental chaos. This contrasts with a dominant theme in the literature, that older adult suicidality typically follows long-standing planning (8). One reason for our findings may be that this study focused on nonfatal suicidal behavior. Planned acts may be more likely to be fatal. Another reason could be shame, given the stigma associated with suicidality; or guilt related to how their suicidal act hurt significant others. In high-income European-descent communities, women and men may have different reasons for describing their suicidal act as unplanned. For women a reason may be that they are socialized to view themselves as emotional and impulsive. For men, a reason may be to distance themselves from a behavior and an outcome (an “attempted” and “failed” suicidal act) that is associated with femininity. In European-descent communities, men are supposed to do suicide “right” the first time, that is, not to survive a suicidal act (see 27–29 for reviews). Given the negative meanings associated with surviving a suicidal act, there may be more variability in older-adult-suicide planning and death intentionality than has been assumed so far. The older-adult suicide planning question should be addressed in larger studies across geographical and cultural settings. Further, the finding that the suicidal state felt like a cognitive and emotional meltdown is consistent with a diversity of evidence derived from studies focusing on younger suicidal persons. Cognitive deficits have been observed in older adults who engaged in a suicidal act as compared to depressed controls who did not (30, 31). The participants in our study were older and had lower ratings of global cognition, compared to the participant of the studies by Crocker and colleagues (2006) and Bonnewyn and colleagues (2014). The age and cognitive characteristics of our study’s participants were more similar to those of participants included in a previous Swedish study (18). In this latter study, 15% of the older adult participants reported having no explanation for their suicidal act. One question is whether late-onset suicidal behavior is a distinct type, with greater cognitive decline and distinct personality traits, for example, rigidity, than earlier onset suicidal behavior (32). In our study, the older adults whose first suicidal act occurred late in life were more likely to report an inability to recognize or adjust to age-related changes, which could suggest rigidity. For those participants the suicidal act was described as something that “happened to them” - emphasizing that their suicidal action occurred in a state of cognitive meltdown. Older adults with a previous history of suicidal behavior may be more similar in personality, coping and mental health history to younger adult individuals who engage in suicidal behavior (32).

A third set of findings is that the participants who reported having had suicidal ideation prior to the act and/or who had insights into its triggers experienced positive changes after the act. Some gained access to good medical care. Others said that they developed increased awareness about their own needs and became more effective at coping. In contrast, participants who said that they had not had suicidal thoughts prior the act and who could not explain their behavior did not report positive changes followed the suicidal act. Although the participants in our study did not directly talk about the act in terms of taking control over their own destiny, there are indication that for some the suicidal act was an attempt to escape a situation of lost control. Studies of control issues in suicide were recently reviewed by Owsiany and Fiske (33). This review concluded that engaging in suicidal behavior as a way to exercise control is relevant to older adult suicidal ideation and nonfatal suicidal behavior. Our study’s results suggest that control and understanding may also be important for recovery.

All but one of the participants were on antidepressants at the time of their suicidal act. Such medication was initiated shortly before the suicidal act in two cases; one participant responded with a manic episode and one with increased anxiety. The debate as to whether antidepressants might increase or decrease the likelihood of suicidal behavior is ongoing. One study found increased suicide probability in young adults who were taking antidepressants, and decreased suicide probability in individuals age 65 and over on antidepressants (34).

### Methodological considerations

This study’s findings should be interpreted in light of the study’s cultural, social and economic context. Overall, the suicide rate in Sweden is lower than the average rate for OECD countries. The 2023 suicide rate was 20.7/100 000 in the 65+ population (women, 11.1/100 000; men, 31.6/100 000) (21). Sweden is a strong welfare state with a public pension system and publicly-funded healthcare. These data indicate that Sweden is a country that provides strong support and resources to its older citizens. Life expectancy is 84.9 years for women and 81.6 years for men. Sweden’s investment in its population has translated in improvements in national health outcomes and high expectations on the part of persons seeking health care. There are also indications of distress in the Swedish older adult population. Antidepressants are widely prescribed to older adults, with substantial differences by sex (women aged 75+, 24%; men 75+, 15%) (35).

A contribution of this study is that it focused not only on what older adults said about what led to their suicidal act but also what changed in them and in their circumstances after the suicidal act. A strength of this study is that it used a two-interview approach. This allowed the participants time for rest and reflection between interviews, and the interviewer time to formulate follow-up questions.

Several methodological features of this study constrain the interpretation of its findings. First, this study sample was small (n=9). Second, it is not known whether the older adults who declined participation differed in systematic ways from those who chose to take part in the study. Third, the research team was mainly composed of professionals with clinical interests and experiences. While this study’s research protocol was designed with a strong focus on the narrative of each participant, the IPA approach is interpretive and builds on the research team’s experiences. The research team’s clinical interest and experiences privileged attention to clinical theories of suicidality and clinical themes in the data. A team with other interests and frameworks (e.g., a gender framework, a socioeconomic framework) could bring to light other themes in the data. Finally, results of our study cannot be directly transferred to settings with other types of health care systems with more limited availability of services.

### Clinical implications

Health care contacts can be suicidogenic as well as suicide-preventive. Some of this study’s respondents described their suicidality as a response to health care professionals they perceived as indifferent and even dismissive of their physical and psychological suffering. In fact, a woman and a man reported that their suicidality resolved once they received the specialist medical care they needed for their physical issues. A potential clinical implication is that continued attention needs to be given to implicit ageism in medical professionals and systems. From this perspective our findings are relevant not only for general practitioners and psychiatrists but also for physicians working in specialist care settings. Older adults are less likely than younger adults to report symptoms related to suicidal behavior, even upon direct questioning (36). Taken together, the findings about older adult suicidality raise questions about the effectiveness with older adults of conventional suicide risk assessment protocols. If the participants in this study had been asked by their general practitioners about their suicidal thoughts, or even about life-weariness, most would likely have reported that they had experienced neither around the time of their suicidal act. What they might have described were feelings of hopelessness, fatigue, resignation and despair about various life challenges. Therefore, it might be more fruitful for professionals working with older adults to focus on life challenges and assess the feelings the older adults have about these challenges – in addition to or instead of conventional suicide risk assessment methods. Another clinical consideration salient to suicide prevention involves restricting means that can be employed in suicide. In our Swedish setting, as in many others, self-poisoning is commonly employed in late-life suicidal behavior (11). This points to the need to better monitor older adults’ prescriptions and use of psychotropic medication.

The study’s participants who lacked comprehension about their suicidal act might be at particular risk of future suicidal behavior. They might benefit from a psychological treatment that focuses on creating a narrative around the suicidal phase. Such treatment could help them regain a sense of control over their own life. A promising treatment is the Swiss-based Attempted Suicide Short Intervention Program (ASSIP) (37). In ASSIP the suicidal person’s narrative is central for understanding reasons for living and decreasing dysfunctional coping (37). The ASSIP model is based on the premise that individual behavior is understandable through the lens of the person’s life history, individual vulnerability, life goals, and basic needs.

Finally, the prevention of late life suicidal behavior should go beyond changes in the health care system. Community-based programs need to be developed for older adults (38). Also, public health interventions that reduce ageism and increase social connectedness are sorely needed.

### Additional directions for future research

Research should continue to focus on the question of why suicide is the way of coping with various adversities, including aging adversities, for some older adults and not most others (28).

In this study some of the narratives of the suicidal trajectory and its aftermath were gendered. For example, challenges to their identity as caregivers in private relationships were highlighted by women, and challenges to their identity as related to mastery in public domains were emphasized by men. Building on gender- and older-adult suicide-scripts theory (39, 28) and evidence (e.g., 40) future research should include questions about gender beliefs and norms of older adult suicidality and examine older adult women’s and men’s narratives in light of these beliefs and norms.

Considering the burden of physical illness and functional limitations that accompany aging, together with the challenges related to the health care system described by all participants in this study, we need to know more about how and why contacts with health care providers may increase rather than alleviate suffering. The role of cognitive impairment in the suicidal process in general, and in the switch from ideation to suicidal action, in particular is another area for future studies.

## Data Availability

The datasets presented in this article are not readily available because the data cannot be sufficiently de-identified to protect the privacy of the participants, making it unsuitable for sharing. Requests to access the datasets should be directed to sara.hed@gu.se.
