# Supplementary material for: Older adults make sense of their suicidal behavior: a Swedish interview study
Source: Front Psychiatry. 2024 Sep 3;15:1450683. doi: 10.3389/fpsyt.2024.1450683 (PMC11413969; doi:10.3389/fpsyt.2024.1450683)
Supplement: Supplementary file 1 [file Table1.docx]

Supplementary Material

| **Supplementary Table 1. Psychotropic medication before and after the suicidal act and at the time of the research interview** | | | |
| --- | --- | --- | --- |
|  | **Ongoing psychotropic medication at the time of the suicidal act** | **Psychotropic medication after the suicidal act** | **Psychotropic medication at time of the research interview** |
| **Participants** |  |  |  |
| **Anna** | Citalopram 20 mg  **Prescription from:**  Primary care  **Length of treatment:**  Many years | Switched to Escitalopram 10 mg, Initiation of Mirtazapine 15 mg, Acamprosate 333 mg  **Prescription from:**  Psychiatric inpatient care | Escitalopram 10 mg,  Mirtazapine 15 mg,  (Acamprosate prescribed but no longer used by participant)  **Prescription from:**  Psychiatric outpatient care |
| **Jane** | Venlafaxin (dosage unclear),  Lithium  **Prescription from:**  Primary care  **Length of treatment:**  Venlafaxin unclear  Lithium, 3-4 years (but discontinued 3-4 months before the suicidal act). | Venlafaxin dosage increased to  150 mg,  Zolpidem 10 mg,  Lamotrigin 100 mg x2,  Oxascand 10 mg (as needed)  **Prescription from:**  Psychiatric inpatient care. | Venlafaxin 150 mg + 37.5 mg, Zolpidem 10 mg,  Lamotrigine 100 mg x2,  Oxascand (as needed)  **Prescription from:**  Psychiatric outpatient care |
| **Emma** | Escitalopram 15 mg,  Mirtazapine 15 mg,  Lamotigrin 50 mg,  Bupropion 150 mg  **Prescription from:**  Primary care  **Length of treatment:** Unclear | Escitalopram 5 mg,  Olanzapin 5 mg x2,  Lamotrigin 100 mg x 2  **Prescription from:**  Psychiatric inpatient care | Escitalopram 5 mg,  Lamotrigin 50 mg 1+0+2,  Olanzapin 5 mg,  Zopiclone 5 mg,  Oxascand 5 mg (as needed)  **Prescription from:**  Psychiatric outpatient care |
| **Cecilia** | Mirtazapine 30 mg,  Zolpidem 10 mg  **Prescription from:**  Primary care  **Length of treatment:**  Mirtazapine 8 years | Mirtazapine 45 mg,  Zopiclone 7.5 mg  **Prescription from:**  Psychiatric inpatient care | Mirtazapine 7.5 mg,  Zopiclone 15 mg  **Prescription from:**  Psychiatric outpatient care |
| **Ben** | Mirtazapine 30 mg,  Oxascand 5mg (as neeeded)  **Prescription from:**  Primary care  **Length of treatment:**  Mirtazapine 30 mg, 1 month prior to the suicide attempt | Escitalopram 10 mg,  Risperidon 1 mg,  Lithium 42 mg (1+1),  Zopiclone 7.5 mg (as needed)  **Prescription from:**  Psychiatric inpatient care | Escitalopram 10 mg,  Lithium 42 mg (dosage is increased to 1+2)  **Prescription from:**  Psychiatric outpatient care |
| **Tom** | Venlafaxin (dosage unclear)  **Prescription from:**  Primary care  **Length of treatment:**  Unclear | Switch to Escitalopram 5 mg, Mirtazapine 30 mg,  Oxascand 10 mg  **Prescription from:**  Psychiatric inpatient care | Mirtazapine 10 mg,  Bupropion 300 mg,  Quetiapin 100 mg  **Prescription from:**  Psychiatric outpatient care |
| **Otto** | None  (Mirtazapine initiated a couple of months before the SA but he stopped taking them after 3 weeks). | Promethazine 25 mg  **Prescription from:**  Psychiatric outpatient care | Mirtazapine 30 mg  **Prescription from:**  Psychiatric outpatient care |
| **Sam** | Sertralin 50 mg (suicidal act 4 days after treatment initiation),  Zopiclone 5 mg  **Prescription from:**  Primary care  **Length of treatment:**  Sertralin 4 days,  Zopiclone unclear | Quetiapin 50 mg  **Prescription from:**  Psychiatric outpatient care | Olanzapin 5 mg,  Duloxitine 60 mg,  Zopiclone 7.5 mg,  Melatonin 5 mg,  Oxascand 5 mg (as needed)  **Prescription from:**  Psychiatric outpatient care |
| **Johan** | Venlafaxin 75 mg  **Prescription from:**  Primary care  **Length of treatment:**  More than 1 year | Venlafaxin dosage increase to  112.5 mg,  Oxascand 5 mg x2,  Zopiclone 7.5 mg  **Prescription from:**  Psychiatric inpatient care | Venlafaxin 75 mg,  Quetiapin 25 mg x2,  Zopiclone 7.5 mg  **Prescription from:**  Psychiatric outpatient care |

**
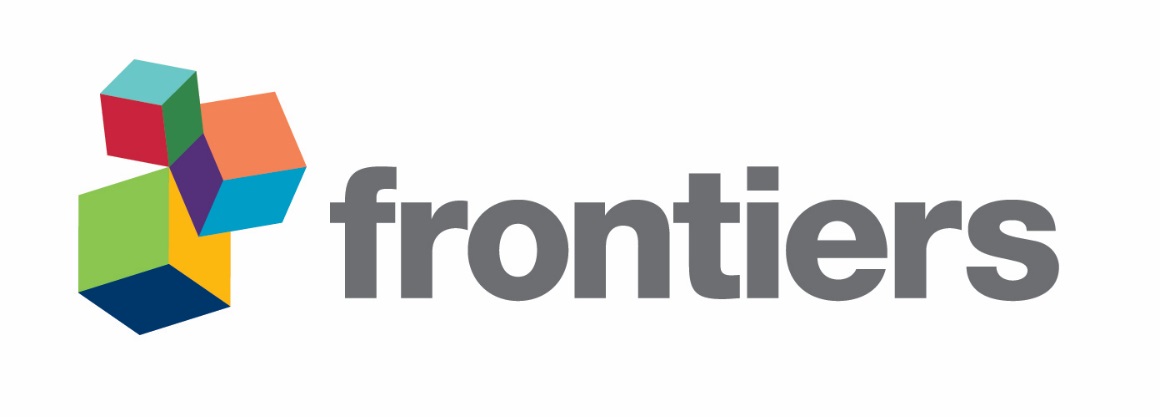
**
